# Internationally Educated Nurses' Experiences of Working in U.S. Long‐Term Care Settings

**DOI:** 10.1002/nur.70034

**Published:** 2025-11-30

**Authors:** Sherif Olanrewaju, Susan J. Loeb, Marie Boltz, Ying‐Ling Jao

**Affiliations:** ^1^ Center for Health Outcomes and Policy Research University of Pennsylvania School of Nursing Philadelphia USA; ^2^ Ross and Carol Nese College of Nursing The Pennsylvania State University University Park Pennsylvania USA

**Keywords:** barriers, facilitators, Internationally Educated Nurses, Long‐Term Care, transition, U.S. Healthcare System

## Abstract

Increasing reliance on Internationally Educated Nurses (IENs) in the U.S. healthcare system, particularly in Long‐Term Care (LTC) settings, necessitates an in‐depth exploration of their transition experiences. The primary purpose of this qualitative study was to understand how IENs describe their experiences transitioning to work with older adults in LTC settings in the United States and the policies and practices that contribute to the successful integration of IENs. A qualitative descriptive design was used, including one‐time individual interviews with 22 IENs working in various LTC settings across the United States via Zoom using a semi‐structured interview guide. Demographic data were analyzed using descriptive statistics, while NVivo 14 software was utilized to organize the data; verified verbatim transcripts were subjected to thematic analysis. Three key themes were identified: Systemic and Practice‐Based Barriers to IEN Integration, Structural and Social Enablers of IEN Integration, and Role of Structured Support Systems in the IENs' Transition. This study highlighted the critical challenges and facilitators that influence IENs during their integration into LTC settings in the United States. Participants reported concerns, specifically noting experiences of racial discrimination and xenophobia perpetrated by co‐workers, patients, and patients' families. These experiences highlight the complex interpersonal dynamics faced by IENs, underscoring the need for proactive strategies to mitigate discriminatory practices and provide culturally sensitive orientation and mentorship programs to support the smooth integration of IENs into LTC settings. Addressing these challenges can have profound implications for strengthening inclusivity and enhancing the overall resilience of the U.S. nursing workforce, particularly within LTC environments.

## 1 Introduction

Internationally Educated Nurses (IENs) are pivotal in addressing nursing shortages in high‐income countries, including the United States. Herein, IENs obtained their nursing licenses in one country (their home country or another) and then relocated to work in another. In the United States., they help fill staffing gaps and strengthen the workforce. According to the Organization for Economic Co‐operation and Development [OECD], the high demand for nurses worldwide has led to a rise in nurse migration, particularly from resource‐constrained to high‐income nations (Socha‐Dietrich and Dumont 2021). Most developed countries address the growing gap between nursing demand and supply by hiring IENs (World Health Organization WHO 2016). Reflecting this trend, the proportion of IENs has increased substantially, from 11% in 2000 to 20% in 2018 across OECD countries (Socha‐Dietrich and Dumont 2021). The primary countries of origin for these nurses include the Philippines, Canada, Kenya, Nigeria, and Ghana (Bakhshi et al. 2025; HRSA 2018). In 2020, the United States was home to approximately 197,000 IENs, accounting for about 36% of the global IEN population (Buchan and Catton 2020). Given these numbers, understanding the integration and employment conditions for IENs in the U.S. healthcare system becomes increasingly critical.

Licensing and registration requirements form the foundation of IEN integration. According to the Commission on Graduates of Foreign Nursing Schools (CGFNS International, Inc 2023), the United States has varied licensing and registration requirements for IENs. These include 1) meeting the basic educational requirements and graduating from an accredited nursing program in their country of education; 2) having credentials evaluated by CGFNS; 3) passing the licensure examination ‐ NCLEX‐RN; and 4) finding a position as a registered nurse. The Nurse Licensure Compact (NLC) allows multi‐state practice without additional licenses, increasing access to employment in other NLC states (National Council of State Boards of Nursing 2025). Streamlined licensure remains essential for effective entry and retention of IENs within the healthcare system.

Immigration policies also influence IEN migration. Policies, such as the 2005 Employment‐Based Immigrant Visa and designation of nursing as a “shortage occupation” (Masselink and Jones 2014) facilitate entry. IENs usually apply under the EB‐3 category for skilled workers (U.S. Citizenship and Immigration Services 2020). Other visa options include the H1‐B Visa for healthcare workers and the TN Visa for Canadian and Mexican citizens under the 1994 North American Free Trade Agreement, primarily used by Canadian nurses (Bakhshi et al. 2025; Villareal and Fergusson 2017). In 2024, nurses accounted for 86% of visa screen certificates (Bakhshi et al. 2025). Navigating these processes often requires assistance from specialized recruitment agencies, underscoring the need for clear and effective immigration policies.

Compounding the critical need for streamlined IEN integration processes is the demographic trend of an aging population in the United States. The number of residents aged 65 and older is projected to reach 82 million by 2030 (U.S. Bureau of the Census 2020), increasing demand for geriatric care. IENs often work in LTC settings, which reflects population trends. IENs must transition smoothly to effectively fill staffing gaps in LTC and provide safe, quality care to older adults. The transition of IENs to their new host countries may be difficult. Ramji and Etowa (2018), using critical social theory and semi‐structured interviews with 14 IENs, found that integration depends on both individual and organizational factors.

To date, no studies have explicitly focused on IENs' experiences transitioning to LTC roles that serve predominantly older adults in the United States, nor on the policies and practices that lead to their successful integration. This study aims to provide a comprehensive understanding of the personal, professional, and systemic factors that influence their integration into U.S. LTC settings and identify key policies and practices that support integration.

### 1.1 Research Questions

What do IENs who provide care to predominantly older adults perceive as the key facilitators and barriers to successful transitions into the U.S. LTC workforce?

What practices and policies support successful transitions for IENs in LTC settings?

## 2 Methods

### 2.1 Study Design

This study employed a qualitative descriptive design to understand the experiences of IENs transitioning to work with older adults in U.S. LTC settings (Sandelowski 2000, 2010; Willis et al. 2016).

### 2.2 Participants

The study population consisted of registered nurses (RNs) who completed their initial nursing education outside the United States and now practice within the country. Inclusion criteria were migration to the United States, employment in LTC, care for adults aged ≥ 65, and current employment as an RN. Exclusion criteria were U.S.‐based RN education, employment outside LTC, and a patient population that excluded older adults. Twenty‐two participants were recruited.

Both purposive and snowball sampling were employed (Pope and Mays 1995; Sandelowski 2000). Recruitment flyers were circulated among ethnic professional nursing associations (e.g., Society of Internationally Educated Nurses in North America [SIENNA]; National Association of Nigerian Nurses in North America [NANNNA]; and the Long‐Term Care Nurses Association). Social media advertisements were placed on Twitter, Facebook, LinkedIn, and Instagram. Additional participants were engaged through the snowball approach.

### 2.3 Screening, Ethical Considerations, and Data Collection Process

Participants who responded to the recruitment flyer were emailed to schedule one‐on‐one interviews. During the Zoom meeting, eligibility questions were reviewed thoroughly. Eligible participants completed a verbal informed consent process, including an opportunity for questions. The Pennsylvania State University Institutional Review Board approved the study.

A semi‐structured interview guide, aligned with the study's objectives, was developed, pre‐tested, and revised with four IENs working in nursing homes (not study participants) to ensure clarity. The guide addressed five sections (I: Preamble; II: Transitioning Experience; III: Preparedness for Working with Older Adults; IV: Workplace Experiences; and V: Reflection and Recommendations). Several items provided participants with lists of potential transitioning experiences (challenges, sources of support), prompting them to elaborate. The complete interview guide is available in Olanrewaju et al. (2025), sections II, IV, and V. The first author, an experienced qualitative researcher and interviewer, conducted the interviews via Zoom. Participants completed a verbal demographic questionnaire on the day of the interview, providing their gender, age, education, country of origin, and work experience. Interviews were recorded with participants' consent, professionally transcribed, and securely stored on the Pennsylvania State University's secure Microsoft Teams platform, which utilizes dual‐factor authentication to ensure confidentiality. Data collection took place in February 2024 and continued until data saturation was achieved. Participants received a $50 electronic gift card for their time.

### 2.4 Data Analysis

The first author completed initial coding and met weekly with the dissertation chair to review progress and address execution challenges. Committee members reviewed a summary document that contained themes, sub‐themes, and quotes from participants. Any inconsistencies that emerged were resolved through team discussions. Demographic data were analyzed using descriptive statistics. Data analysis was supported by NVivo, allowing for systematic coding and thematic development. Following Braun and Clarke's (2019) six‐stage thematic analysis process—familiarization, coding, theme generation, review, naming, and writing—the researcher employed deductive and inductive approaches. Deductive analysis provided structure, while inductive analysis allowed themes to emerge naturally without preconceived expectations. Transcripts were read multiple times to deepen familiarity with IENs' experiences. Data were broken into text segments, each reflecting a specific idea related to the research questions. For example, one participant's account of racial abuse was coded as “racism and xenophobia.” In total, 12 initial codes were developed and grouped into three broader themes. Themes were obtained by grouping similar codes into broader categories of meaning (Braun and Clarke 2019). Each theme was then aligned with the research questions to determine its relevance.

Findings were structured around the research questions, with Table 1 showcasing the theme‐naming process with additional quotes from participants.

**Table 1:** Naming of themes.

Theme	Theme definitions	Subtheme	The number of participants contributed	Additional Respondent quote
Systemic and Practice‐Based Barriers to IEN Integration in Long‐Term Care	Difficulties and barriers that IENs face while adapting to a new work environment	Racism and Xenophobia	Eighteen	“The prejudice is there. They [patients and their families] think, oh, she's Black, she doesn't know what she's doing… there are times that probably you have like three white people on the floor and probably two Black people. They will pass those two Black people and talk to the White people and go and ask for the nurse or the CNA thinking that the CNA is a nurse. So, there is this prejudice that Blacks are just carers. They can't be nurses. (Nigeria, female).”
				“I think America as a nation has had this long history of racism and stigmatization. I feel like being a Black person is number one [a stigmatized identity], and being a Black immigrant is also another one. It is like two [stigmatized identities]. That is always there. It is always there. It is very interesting that you are taking care of these patients living with dementia, and some of them use racial slurs at you. I have had experiences like that. I have seen some say, “Go back to your country. What are you doing in America? (Nigeria, female).”
		Language and Cultural Barriers	Eighteen	“There are accents I can definitely not understand over here. So, I keep on having to tell someone to repeat. And not everyone is willing to understand that, and maybe do it like repeat or let me get what they are saying. And also, there are people who be like, ‘Oh I don't understand what you're saying,’ so they might not want to work with me. (Kenya, female).”
				“I'm Zimbabwean, we don't like complaining a lot. But one thing I've discovered is in America if you keep quiet, you are satisfied. [Laughs.] Those are the things I'm learning by just living in America. You have to be vocal, to speak when you need to speak (Zimbabwe, female).”
		Credentialing	Eleven	“I am facing a lot of problems in terms of accreditation. They were not very receptive to getting accredited my degree and getting me enrolled as a clinical nursing training. It took, I think, nearly four to five months just to get accredited. They [U.S. nursing boards] have their own paperwork, and they had to verify the documents back to India as well. It was a very challenging experience. (India, male).”
				“The fact I got my New York license was a problem. It took me like a year to get the nursing [license]. New York was so slow… you call them, call them, call them. They put you on the phone [on hold] for a long time. (Nigeria, male).”
		Socio‐Environmental Stressors	Fourteen	“When I moved here my daughter just turned 18 when they were filing, just before they filed for here so they couldn't file for her. That really stressed me out. Up to now it affects me because I couldn't come with her. (Zimbabwe, female).”
				“…definitely the weather. It's not what I'm used to because in the Philippines, we have a really warm – it's mostly warm and small days where we get rain. But it's a tropical country so, all around the year, it's hot. And in here, we get winter and I'm not used to that so, it's a big challenge for me to work when there's snow, yes. And that's what my concern is. (Philippine, female).”
Structural and Social Enablers of IEN Integration in Long‐Term Care	Supportive factors or resources that assist internationally educated nurses in adapting smoothly to a new work	Previous Experience Aided Transition	Nineteen	“The education in the Philippines, they're very strict, especially with time management. And yeah, it helps a lot, because we are trained from the beginning in how to do time management. (Philippine male)”
				“The kind of teachings we had back home are the same thing here. That really helped a lot. Nigeria female”
		Support Network	Eighteen	“I'm happy that I have friends who have already immigrated into America who are also supportive in telling me, ‘No, no, no, you made a mistake by doing this. Why not do this? Oh, next time you should do this, next time call me. These are supported systems that helped me through this process. (Nigeria, female)”
		Mentoring and Peer Support	Thirteen	“When I started, I really got support from my colleagues at the time. And when I started work, I had to tell my DON that I'm going to be a nurse here for the first time. I haven't worked in these settings at all, so I needed more time. So, I didn't want to be a supervisor until I know what I'm doing. And during that time, the DON really supported me. I was given more time of training. (Kenya, female)”
		Orientation and Training	Ten	“The first facility that I went to as a staff nurse was a very good orientation that they gave us. The director of nursing at that time was very involved with the staff. There were meetings all the time. Even the administrator was very supportive and welcoming. It was a nice place to work. (Philippine, female)”
				“I think one of the things that makes me successful in this transitional phase is the orientation program… I was assigned a preceptor that watched over me, that guided me and oriented me to the facility. When I came out of that orientation, it was great. (Nigeria, female)”
Role of Structured Support Systems in IENs Transition	Organizational guidelines and practices designed to support IENs in effectively integrating into the workplace	Nurse Licensing Compact	Fifteen	“By the time you want to endorse your license to another state, that is when it is always a pain. It is very difficult. It is like starting all over again. That is the process. I have a colleague of mine who chose to move to Georgia.… I asked her if she is now practicing. She said no. She has to do the whole CGFNS (Commission on Graduates of Foreign Nursing Schools) processing again. (Nigeria female)”
		Mentoring from supportive managers	Eight	“One of the successful programs during my transition period is a nurse mentoring program. It is very applicable for me being an international nurse here in the United States of America. I am seeing it as very important, actually. That is an external program. That is not under my employer's listed functions. There were programs offered by external partners or external institutions which were a big help for me. (Philippine, male)”
				“I am currently a part of their mentoring program. I am a mentee under the Philippine Nurses Association of America. Also, I am currently under the nurse mentoring program of the American Nurses Association. I considered it a great help for me in dealing with my RN and doing my transition or adaptation here in the US. My mentors taught me how to deal with those employers that treated me unfairly with work. I gave my letters. I cannot say it is a complaint, but it is a paper trail of what they did to me. They told me what to do and what organizations should have filed those paper trails. (Philippine, male)”

This study employed multiple strategies to ensure the trustworthiness of the findings (Lincoln and Guba 1985). We ensured credibility through persistent observation, active listening, and analytic attention on domains most salient to the research questions. Sufficient engagement with participants helped build rapport, learn contextual norms, and verify information through repeated contacts and iterative member checking. A member check was conducted by sharing the primary themes of the study with eight participants who expressed a willingness to provide feedback. Five participants responded with feedback. The interviewer engaged in active bracketing throughout the research process to minimize the influence of personal biases and assumptions. As part of his positionality (Husserl 1962), he recognized his identity and prior experiences as an IEN, acknowledging that these could shape the interpretation of participants' narratives. To manage this, he maintained a reflexive journal throughout the data collection and analysis, as part of the bracketing process. Transferability was enhanced by providing detailed descriptions of the research site, participant demographics, and methods. Dependability was maintained through consistent data collection by the same investigator using the same interview guide across all participants. Data analysis and collection occurred concurrently, enabling the refinement of interview questions as new themes emerged. Confirmability was strengthened by cross‐referencing interview notes with transcripts, grounding findings in participant narratives rather than the researcher's preferences. Authenticity was upheld by fully briefing participants on the study, addressing their questions, and ensuring confidentiality and privacy. This approach allowed participants to express their views concerning their transitioning experiences candidly.

## 3 Results

### 3.1 Demographic Characteristics of Participants

Twenty‐two participants represented eight countries: Nigeria (*n* = 9), the Philippines (*n* = 5), Kenya (*n* = 3), and one each from India, Jamaica, Ghana, South Korea, and Zimbabwe. They worked across seven states: Maine, Missouri, Maryland, New York, Illinois, Texas, and Pennsylvania. Ages ranged from 30 to 57, with a mean of 40.45 years (±7.67). Most were female (82%) and married (68%). All participants, except one Kenyan educated in the U.K., completed their nursing education in their home countries. The highest degree achieved was a Master of Science in Nursing (MSN), and most participants (73%) held a Bachelor of Science in Nursing. Over half (*n* = 12; 55%) had 2–5 years of working experience in U.S. LTC.

#### 3.1.1 Research Question One

What do IENs who provide care to predominantly older adults perceive as the key facilitators and barriers to successful transitions into the US LTC workforce? Two major themes emerged.

##### 3.1.1.1 Theme 1: Systemic and Practice‐Based Barriers to IEN Integration in Long‐Term Care

Participants identified key challenges, including Racism and Xenophobia; Language and Cultural Barriers; Credentialing; and Socio‐environmental Stressors. All 22 participants contributed data to this theme.

###### 3.1.1.1.1 Subtheme 1a: Racism and Xenophobia

Eighteen participants highlighted significant encounters with racism and xenophobia, often from residents under their care. Many described experiences of explicit verbal hostility and instances of rejection rooted in racial prejudice or misconceptions about their cultural identity. P15 recounted direct confrontations from residents, who openly questioned her presence and capability, stating, *“Somebody will ask you like, ‘Why did you come here? Why don't you go back to your country?” Some of them don't want to be taken care of by you.” (Female, Kenya)*. Such remarks reflect explicit xenophobic sentiments, highlighting a distinct rejection of care based solely on nationality and ethnicity. Moreover, the hostility encountered extended beyond verbal abuse into physical aggression and derogatory misidentification. P2 described his experience facing verbal abuse (e.g., discriminatory slurs and hostile remarks) from residents:

I am new here and also, I belong in another culture, they sometimes say words that are discriminatory and derogatory. They try to hurt me in a little way. I experience verbal abuse. They are cussing me because I am new here. They are saying that I am Chinese. I am not Chinese. I am Filipino.

(Philippines, Male)

His experience underscores the layered complexity of xenophobia intertwined with racial stereotyping.

Participants also reported experiences of racial discrimination from residents' families, which impacted their professional interactions and diminished their perceived competence. P3 described explicit racial bias exhibited by patients and their families, emphasizing how race‐based assumptions affected their professional credibility:

The prejudice is there. They [patients and their families] think, oh, she's Black, she doesn't know what she's doing… there are times that probably you have like three white people on the floor and probably two Black people. They will pass those two Black people and talk to the White people. So, there is this prejudice that Blacks are just carers. They can't be nurses.

(Nigeria, female)

Participants faced stigma and discrimination not only for being IENs who self‐identify as Black but also for their immigrant status, leading to layered abuse and marginalization from the residents they cared for.

Additionally, discrimination was not limited to interactions with patients and their families but occasionally extended into workplace dynamics among immigrant colleagues. One participant shared that a senior immigrant colleague conspired with supervisors to assign her complex tasks and block bonus shifts. Another recounted a toxic relationship with a fellow migrant colleague, initially perceived as a friend but later viewed as hostile: “I thought he was a friend the first day I was introduced to him, but as time went on, I actually discovered this is my worst enemy” (Zimbabwe, Female). Collectively, these accounts reveal complex layers of racism and xenophobia experienced by immigrant nurses, manifesting through overt aggression, subtle biases, and interpersonal conflicts that cumulatively pose significant emotional and professional challenges.

###### 3.1.1.1.2 Subtheme 1b: Language and Cultural Barriers

Eighteen participants identified significant language and cultural differences that complicated their transition into U.S. LTC settings. Language barriers included accent‐related communication difficulties, misunderstandings of medical terminology, and challenges expressing themselves professionally. P10 stated: “*Communication barriers come into play [sic]. It is so hard… Working with other staff, even communicating what you need from your coworkers, sometimes it is hard for them to understand.”* (Philippines, female).

These barriers caused both practical difficulties and emotional strain.

Cultural barriers often intersect with language challenges, influencing participants' interpersonal interactions and professional identity. For example, several participants noted stark contrasts in how nursing is valued. P1 shared:

There was a great culture shock for me. Where I'm coming from, they see nurses as the little gods. I think the reverse is the case here… They don't appreciate the efforts of nurses. Whatever you do to them, they believe it's just their right. And you go extra miles to make sure they are comfortable.

(Nigeria, female)

This reflection highlights broader implications for the professional esteem of immigrant nurses. Additionally, participants identified cultural expectations around family care as another key barrier.

Others highlighted disparities in societal norms around older adult care, notably the shift from collective familial care to a more individualized and institutionalized model in the U.S. healthcare system.

My culture is very family‐centered. So, especially if an older person is sick, their child and their other family members, they're going to take care of the patient. But here, it's a huge difference. They are more individualized. So, they rely on the government. They were focused on themselves.

(South Korea, female)

Such insights illustrate the deeper cultural disconnect participants faced, necessitating not only adjustments in practice but also significant adaptations in their personal values and expectations.

###### 3.1.1.1.3 Subtheme 1c: Credentialing

Eleven participants described credentialing as a significant barrier, citing delays, bureaucratic complexities, and high costs. The process involved multiple layers of approval, extensive documentation, and substantial financial investments. P12 captured these difficulties, recounting how the process extended across multiple countries and involved considerable financial hardship:

During my transition stage, it really took a lot of time because before you can practice in the United States, you must meet their criteria. So, I have to pass through a lot of hurdles. Having to register with my Board of Nursing, having to pass my NCLEX exam [National Council Licensure Examination] as a matter of fact…This really took a lot of time because back home in Nigeria, we don't have an NCLEX exam center, so I have to travel as far as South Africa to have my NCLEX written… So, it took a lot of time. And when you talk of money, it is capital intensive… my pocket was drained.

(Nigeria, female)

Moreover, participants emphasized how even minor administrative errors caused significant setbacks, exacerbating frustration and emotional distress. P11 described how a simple clerical mistake by the Nigerian Nursing Board on her birthdate caused long delays and intensified her sense of helplessness:

… It took them a long time [to correct it]. It was so frustrating. I lost hope and everything.

(Nigeria, female). Such incidents not only delayed professional integration but also took an emotional toll.

###### 3.1.1.1.4 Subtheme 1d: Socio‐Environmental Stressors

Fourteen participants shared challenges related to loneliness and unfavorable weather conditions. Many experienced emotional isolation, which negatively impacted their integration. During her interview, P17 became visibly distressed when discussing life in the United States without her family. Although the researcher offered a referral for support services, she declined, stating she did not need additional support. The transition process has evidently caused profound disconnection for IENs from their families. The experiences of several participants are reflected in their narratives. P18 shared: “*I started here all by myself. It was very difficult because you must deal with everything. I really miss home big time. I went home, got married, and my husband came here. It was a lot better”* (Philippine, female).

P4 recounted similar issues:

I have parents and we are a nuclear family, so I had to separate, and I had to move here…I would say it was a lonely experience. I would say it's quite lonely conditions than in my country [sic].

(India, male)

In addition to family separation and loneliness, some participants described struggling to adapt to the cold weather in various parts of the United States, which contrasted with their warmer home climates and affected their personal and family lives. P11, who transitioned to Indiana from Zimbabwe, found the cold compounded her sense of isolation, while P12 and her family faced similar challenges transitioning from Nigeria. “*When we came to the United States, it was extremely cold. So even my children had a hard time adapting initially to having to wear thick clothes. …weather was a big challenge when we came.”* (Nigeria, female).

Participants from tropical climates, like the Philippines, struggled with snow and harsh winters, which affected both their work and personal life. These experiences highlight the participants' environmental adjustment and personal resilience in balancing professional transitions, adapting to a new climate, and managing distant family responsibilities.

##### 3.1.1.2 Theme 2: Structural and Social Enablers of IEN Integration in Long‐Term Care

All 22 participants contributed data to this theme by describing their transition to U.S. LTC settings with respect to the facilitators they experienced.

###### 3.1.1.2.1 Subtheme 2a: Previous Experience Aided Transition

Nineteen participants reported that premigration nursing experience eased their transition. They highlighted the universality of nursing knowledge and skills, indicating that foundational nursing practices were consistent globally. P1 reflected, *“I would say nursing is the same everywhere. The kind of teachings we had back home are the same thing here. That really helped a lot.”* Thus, the universality of nursing skills was a facilitator of her transition because she did not need to relearn her profession.

Several participants described how previous experience supported their transition. For example, P14 noted that her extensive background in trauma and emergency care provided her with the critical decision‐making skills needed to respond effectively in emergent situations within LTC environments. She explained, “*I did more of trauma while I was in Nigeria…working in trauma did prepare me in the sense that, that sense of urgency, to take a decision, this person needs more advanced healthcare.”* (Nigeria, female). Similarly, P15 identified her prior experience with elderly patients as particularly beneficial, stating, *“Most of the time I worked with the older patients… So, that helped me a lot, because I knew how to deal with the with the older patients.”* (Kenya, female). These experiences significantly reduced the participants' learning curve and enabled a smoother professional and emotional transition into their new roles.

###### 3.1.1.2.2 Subtheme 2b: Support Network

Eighteen participants reported that support from their friends and families was an important facilitator of their transitions. P14 said in a representative response that during her transition, *“Having my family around was a very big plus for me… it was a good support system for me. And that made a lot of difference.” (Nigeria, female)* In another representative response, P3 described the family as her most important support: “*My family was the biggest support I had throughout the process. It wasn't an easy process for me, but with the kind of family I had, and the kind of support, they made the journey so smooth for me.”* (Nigeria, female).

P3 relied on her husband, parents, and daughters as social support during her transition. Most participants, particularly Filipinos and Nigerians, the two largest IEN groups in the United States, reported that family and immigrant friends helped to facilitate their transition. Furthermore, professional associations like the SIENNA and NANNNA provide essential social support and networking opportunities.

###### 3.1.1.2.3 Subtheme 2c: Mentoring and Peer Support

Thirteen participants emphasized how mentorship and guidance from experienced colleagues and supervisors eased their integration. P12 expressed deep appreciation for the openness and accessibility of colleagues who willingly provided practical advice, stating,

When it came to the support I got from my colleagues, they were of immense help. … If there is any way I need to seek clarification, I don't hide, I tell them: ‘Oh, can you tell me how to do this?

(Nigeria, female)

Such peer interactions were pivotal in creating an environment of collaborative learning and psychological safety. Additionally, some participants described supervisors who supported not only their professional roles but also their personal and family needs. P14 described a particularly supportive manager whose proactive approach significantly impacted her family's wellbeing, sharing,

We had a manager that even though she was not an international nurse, she had worked with certain international nurses. She would ask you questions like, ‘Hope your children are settling good into their schools.” Oh, your spouse, I hope he has a job’. Like, my manager worked on it and found a job for my spouse. She was a good support system financially, psychologically, and otherwise.

(Nigeria, female)

P14's experience highlights the importance of supervisors who recognize the broader social and familial factors that influence nurse performance. Collectively, these narratives demonstrate how mentoring and peer support were crucial in fostering professional competence and emotional resilience during the transition.

###### 3.1.1.2.4 Subtheme 2d: Orientation and Training

Ten participants described that structured orientation and training were important to their successful transition. Many described the onboarding process as supportive, structured, and tailored to their individual learning needs. P8 highlighted the effectiveness of a supportive preceptorship during her orientation, stating,

I let the management know that it was my first time working in the U.S. as a nurse. They were patient with me. The onboarding process was hitch‐free. I had a very good preceptor that was very patient in helping me transition and get full knowledge of whatever I was going to do. That helped a lot.

(Nigeria, female)

Her comment reflects the broader sentiment that individualized training contributed significantly to easing their professional adjustment.

Furthermore, participants noted the value of nurse educators who are explicitly assigned to support IENs. P17 described how visiting nurse educators collaborated closely with her mentor to customize the orientation process to address her specific needs:

Educators who were responsible for international nurses would come on the unit and ask, ‘How are you getting along?’ have a meeting with you and your mentor to see what's going on, what can they do to help? Based on what you're experiencing, they were able to adjust their orientation package.

(Jamaica, female)

This tailored approach reinforced participants' confidence and promoted inclusion and support.

However, three participants reported discrepant experiences, indicating issues related primarily to communication difficulties and managerial interactions. For instance, P1 described her frustration with a manager who consistently exaggerated language barriers related to her accent, noting, *“I had a hard time with my manager because she pretends, she doesn't understand what I'm saying. She always says, ‘Huh? Huh? Huh?’ I had to write down a lot of times.”* (Nigeria, female). Nonetheless, such experiences were not widely reported; instead, most participants viewed their orientation and training as facilitators in their transition into the U.S. LTC settings.

#### 3.1.2 Research Question Two

What practices and policies best support transitions for IENs who provide care to predominantly older adults? One theme emerged during data analysis to address this question.

##### 3.1.2.1 Theme 3: Role of Structured Support Systems in IENs Transition

Twenty‐one participants described policies and institutional practices that supported successful transition, including the NLC; comprehensive orientation programs and onboarding programs, as discussed under Theme 2 (facilitators); and structured mentorship.

Fifteen participants specifically described the NLC as significantly beneficial in streamlining credentialing across states. For instance, P11 expressed appreciation for the compact's practicality: *“It really helped me because when I went to Indiana, I got a Compact license from the Indiana Board of Nursing, and that's the license I'm still using to work in Texas.”* (Nigeria, female). P12 recommended expanding the number of states involved in the Compact*: “I was faced with the hurdles of having to go through the endorsement process. So, I think if we have more compact states, this is going to help for the smooth transitioning process.”* P14 said that the compact was so helpful that she advised other IENs to obtain a compact license. P6 also described the significance of the NLC in facilitating the ease of relocation between states.

By the time you want to endorse your license to another state, that is when it is always a pain. It is very difficult. It is like starting all over again. …If we are moving from State A to State B, we should not have to cross oceans to get another license in another state.

(Nigeria, female)

Participants described the NLC as helping to ensure a smooth transition.

Twelve participants identified orientation and onboarding processes as crucial, structured practices that enhance their transition. Participants articulated the importance of tailored, extensive orientation programs provided either directly by employers or employment agencies. P1, for example, detailed her orientation experience, emphasizing its comprehensive nature:

When coming from Nigeria, the agency was there for us. They put us in a place and trained us for 4 weeks, changing our orientation [according to] where we're coming from and what is expected of us at the place of work, I think I had about 6 weeks orientation as well. Those were helpful.

(Nigeria, female)

Also, P20 described a helpful onboarding program through his employment agency: “*I felt the onboarding program was all‐encompassing. It brought everything together into one…., they let you know what is expected of you.”* (Ghana, male). Thus, participants found these programs effective in promoting their successful transition.

Additionally, structured mentorship emerged as a critical factor for professional skill‐building as well as emotional and social support, cited by eight participants. P17 valued mentorship particularly due to shared cultural backgrounds: *“I was lucky enough to have a Jamaican as my mentor. Whenever I wasn't up to par with certain things, she was there to make sure that I was up to standard” (Jamaica, female)*. Similarly, P3 received mentorship from a preceptor who is a fellow IEN employee.

My preceptor was in charge of my orientation stage, and even after the orientation, it was really an easy process for me because I could go to them to ask questions. If I had any difficulties, I could easily go to them and ask questions. They made it very, very easy for me.

(Nigeria, female)

P2 acknowledges the significance of mentorship, not solely for professional development, but also as a vital support mechanism in navigating personal, social, and cultural adaptation processes. Overall, participants identified mentorship as a best practice for their successful transition.

## 4 Discussion

This study aimed to understand the experiences of IENs transitioning into U.S. LTC settings and identify key policies and practices that support integration. Findings revealed major barriers and facilitators that impact IENs' experiences. Participants frequently identified racism and xenophobia from co‐workers, patients, and patients' families, including verbal abuse, and rejection of care by residents based on their race or immigrant status. These experiences were notably intensified for IENs who self‐identify as Black, aligning with previous research indicating pervasive prejudice against racial minorities in healthcare settings (Jenkins and Huntington 2016; Ham 2021). ZareKhafri et al. (2022) identified organizational‐cultural discrimination as one dimension of discrimination against ethnic‐cultural minorities. In contrast, this study also revealed discrimination among migrant nurses of the same ethnic‐cultural group. Given these troubling findings, it is imperative that LTC administrators implement structured antidiscrimination policies, cultural sensitivity training, and clear reporting protocols. Training should include real‐world scenarios to prepare staff and to address intra‐group discrimination among migrant nurses effectively.

The study highlighted language and cultural barriers that complicated IENs' ability to deliver effective care. Participants noted difficulties communicating with coworkers and residents due to accent differences, language proficiency gaps, and cultural expectations. Since LTC residents often have chronic conditions, cognitive impairments, and unique psychosocial needs, IENs had to adapt quickly to person‐centered care while navigating communication gaps. These findings resonate with prior research linking communication barriers to medication errors, miscommunication, and ineffective care (Wolcott et al. 2013; Eriksson et al. 2023). Cultural differences in practices, norms, and nursing expectations create challenges for IENs, leading to workplace conflicts. Prior research supports these findings, showing that IENs working abroad often encounter challenges related to varying beliefs, customs, and behaviors (Iheduru‐Anderson and Wahi 2018; Merry et al. 2021; Rosenkoetter et al. 2017). Nursing administrators should develop training programs that promote cultural competence and include advanced language support, informal conversation practice, and education on culturally sensitive care. Training should also reflect the communication challenges identified by participants, such as accent comprehension and culturally influenced expectations of nursing roles.

Credentialing was another significant barrier. Participants described the credentialing process as bureaucratic, time‐consuming, and costly. Verifying foreign qualifications, undergoing additional training, and taking mandatory exams exacerbated stress and delayed integration into U.S. LTC settings. These findings support previous research by Dahl and colleagues describing credentialing as exhausting and inefficient (Dahl et al. 2022). Policymakers should develop more streamlined, transparent credentialing systems and expedited pathways that recognize international experience, qualifications, and the diverse educational backgrounds of IENs.

The study identified mentorship, orientation, and licensing flexibility as important facilitators of successful IEN integration. Participants highlighted mentorship as pivotal for professional development and personal adaptation. Formal programs pairing IENs with experienced LTC nurses can enhance retention and professional confidence. The study aligns with previous research, emphasizing that curriculum improvement, mentoring, and professional development prepare nurses to care for older adults (Abudu‐Birresborn et al. 2019). LTC facilities should implement formal mentorship programs that offer regular feedback, professional development, and psychosocial support. Orientation and onboarding processes were also critical, particularly when tailored to IENs' educational and cultural backgrounds. Participants valued comprehensive orientation programs from employers or recruitment agencies, which prepared them for the demands of LTC settings. Facilities should develop standardized yet adaptable onboarding curricula, informed by the findings from this study, incorporating training on clinical protocols, patient‐centered care, communication, and cultural expectations. Finally, participants strongly endorsed the NLC for easing their professional transition and facilitating inter‐state workforce mobility. Expanding the NLC to more states and enhancing policy advocacy efforts should be prioritized, aligning with recommendations from the Federal Trade Commission (Federal Trade Commission 2017). The findings suggest that mentorship, orientation, and licensing flexibility are key to successful IEN integration into U.S. healthcare.

As more nurses migrate from resource‐constrained countries to high‐income nations, fair and supportive policies are needed to protect healthcare workers and benefit both source and destination countries. The U.S. healthcare sector faces rising RN demand amid shifting immigration policies and visa uncertainty, which could hinder efforts to address RN shortages and meet workforce demands (Bakhshi et al. 2025). This study's implications underscore the urgent need for targeted interventions, robust antidiscrimination policies, and comprehensive training programs to support IEN integration into LTC. Further intervention‐based research should evaluate and standardize strategies that address the challenges revealed by participants. Ensuring successful integration of IENs will enhance care quality, foster workplace inclusivity, and benefit both healthcare providers and recipients in increasingly diverse U.S. LTC environments.

## 5 Limitations

The study's limitations include its focus on a small sample of IENs, though data saturation was achieved. Expanding the sample to include a more diverse and representative group could improve the generalizability of the findings, especially regarding national origins, gender, and work settings. The findings may not be applicable to other migrant healthcare professionals in different U.S. healthcare settings. Furthermore, only IENs who had been in the United States for at least 1 year were included, possibly excluding those with more challenging transitions who left earlier, which may affect the validity and transferability of the findings.

## 6 Conclusions

This study has provided significant insights into IEN's experiences transitioning to work in LTC settings. Facilitators were identified that promote the smooth transition of IENs into the United States, including support networks from family and friends, mentorship, and focused training on caring for older adults. The healthcare system in the United States requires adopting strategies, such as the Nurse Licensure Compact, to facilitate the easier transfer of licenses between states. Additionally, orientation and onboarding, as well as mentorship, are crucial.

## Author Contributions

**Sherif Olanrewaju:** conceptualization, methodology, data curation, formal analysis, investigation, writing – original draft, writing – review and editing. **Susan J. Loeb:** conceptualization, methodology, formal analysis, writing – review and editing, supervision. **Marie Boltz:** conceptualization, methodology, writing – review and editing. **Ying‐Ling Jao:** conceptualization, methodology, writing – original draft, writing – review and editing.

## Conflicts of Interest

The authors declare no conflicts of interest.

## Data Availability

The data that support the findings of this study are available from the corresponding author upon reasonable request.
